# Perceptions of physicians caring for pediatric patients with cancer in Europe: insights into the use of palliative care, its timing, and barriers to early integration

**DOI:** 10.3389/fonc.2024.1461668

**Published:** 2024-10-23

**Authors:** Ana Lacerda, Isabel M. Bravo-Carretero, Bella S. Ehrlich, Godwin Job, María Avilés Martínez, Ulrike Leiss, Georgia Kokkinou, Katrin Scheinemann, Finella Craig, Kerstin Krottendorfer, Meenakshi Devidas, Justin N. Baker, Asya Agulnik, Michael J. McNeil

**Affiliations:** ^1^ European Society of Paediatric Oncology (SIOP) Europe Palliative Care Working Group Steering Committee, Brussels, Belgium; ^2^ European Association for Palliative Care Children and Young People Reference Group Steering Committee, Vilvoorde, Belgium; ^3^ Department of Paediatrics, Portuguese Institute of Oncology, Lisbon, Portugal; ^4^ Universidad Central Del Caribe, School of Medicine, Bayamón, Puerto Rico; ^5^ Brown University, School of Medicine, Providence, RI, United States; ^6^ Department of Global Pediatric Medicine, St. Jude Children’s Research Hospital, Memphis, TN, United States; ^7^ Paediatric Palliative Care Unit, Niño Jesús Children’s University Hospital, Madrid, Spain; ^8^ Department of Nursing, Autonomous University of Madrid, Madrid, Spain; ^9^ Department of Paediatrics and Adolescent Medicine, Medical University of Vienna, Vienna, Austria; ^10^ Floga-Parents Association of Children with Cancer, Athens, Greece; ^11^ Childhood Cancer International Europe, Vienna, Austria; ^12^ Division of Oncology/Haematology, Children’s Hospital of Eastern Switzerland, St. Gallen, Switzerland; ^13^ Faculty of Health Sciences and Medicine, University of Lucerne, Lucerne, Switzerland; ^14^ Department of Paediatrics, McMaster Children’s Hospital and McMaster University, Hamilton, ON, Canada; ^15^ Louis Dundas Paediatric Palliative Care Centre, Great Ormond Street Hospital, London, United Kingdom; ^16^ Division of Quality of Life and Pediatric Palliative Care, Stanford University, Palo Alto, CA, United States; ^17^ Division of Quality of Life and Palliative Care, Department of Oncology, St. Jude Children’s Research Hospital, Memphis, TN, United States

**Keywords:** palliative care, pediatrics, oncology, education, services

## Abstract

**Background:**

Integrating pediatric palliative care (PPC) into pediatric oncology standard care is essential. Therefore, it is important to assess physicians’ knowledge and perceptions of PPC to optimize its practice.

**Objective:**

To evaluate the knowledge, comfort levels, and perspectives of physicians regarding the timing and perceived barriers to integrating PPC into pediatric cancer care across Europe.

**Design:**

The Assessing Doctors’ Attitudes on Palliative Treatment (ADAPT) survey, originally developed for other global regions, was culturally and contextually adapted for Europe.

**Setting/Subjects:**

The survey was distributed via the European Society of Paediatric Oncology (SIOPE) membership listserv. Any physicians caring for children with cancer across Eastern, Southern, Central, and Northern Europe were invited to complete the survey.

**Results:**

A total of 198 physicians from 29 European countries completed the ADAPT survey. Physicians demonstrated relative agreement with the World Health Organization’s guidance; median alignment was 83.4% (range 59.9%-94.1%). Although most respondents felt comfortable addressing physical (84.4%) and emotional (63.4%) needs, they felt less comfortable addressing spiritual needs (41.9%) and providing grief and bereavement support (48.5%). There were significant regional differences, such as physicians in Eastern and Southern Europe reporting a lack of PPC specialists, opioids, and home-based care, while those in Northern and Central Europe did not.

**Conclusion:**

Physicians caring for children with cancer throughout Europe have a good understanding of PPC. However, misconceptions about PPC persist, requiring educational and capacity-building efforts. Additionally, the regional differences in perceived barriers must be addressed to ensure equitable access to PPC for all European children with cancer.

## Introduction

The World Health Organization (WHO) has defined palliative care (PC) as “an ethical responsibility of health systems, regardless of resources.” (1) The need for PC is particularly important for pediatric populations. Evidence suggests that early integration of palliative care for children with cancer and other serious illnesses has significant benefits for patients and their families, including improved pain and symptom management, reduced caregiver and patient suffering, and better family communication (2–5).

An estimated 21 million children worldwide require pediatric palliative care (PPC) each year, with 8 million needing specialized PPC services (6). Children with cancer comprise a distinct cohort within this demographic, not only due to the symptoms and stress of having cancer, but also treatment-related toxicities they endure (7–11). In 2018, the WHO launched its Global Initiative for Childhood Cancer, with the goal of increasing pediatric cancer survival to 60% by 2030, while also alleviating suffering and improving the quality of life for children with cancer globally (12).

Unfortunately, the development and access to PPC lag far behind those of adult-oriented services (2). This discrepancy is due to multiple factors, including geography, lack of education, and a general lack of public awareness about PPC (2, 13–15). Other common barriers to integrating PPC into pediatric cancer care include physicians’ perceptions about the role and timing of PC, and the emotional relationship between the oncologist and their patients and families (16–19).

The WHO called for the strategic planning and implementation of PPC initiatives tailored to the local needs and opportunities of each region or country (2). Europe is a heterogeneous region, comprising 50 countries that communicate in 24 official languages (20, 21). Childhood cancer is the leading cause of disease related death in children older than 1 year of age in Europe with an estimated 24,000 new cases each year (22). Economic growth and development also vary greatly, from some of the world’s wealthiest countries to low- or middle-income countries (LMICs) (23–25). These disparities contribute to the variability in and accessibility to PPC services (26, 27). To address the need for PPC provision for children with cancer in Europe and reduce inequities in quality of life across regions, the Board of the European Society for Paediatric Oncology (SIOPE) recently established a PPC Working Group (7).

Furthermore, the Assessing Doctors’ Attitudes on Palliative Treatment (ADAPT) survey was developed to assess the perceptions of physicians who treat children with cancer about PPC timing and integration in their setting (28–33). In collaboration with the SIOPE PPC Working Group, we distributed this survey in Europe (ADAPT-E) to learn physicians’ perceptions about integrating PPC into standard care for children with cancer in SIOPE member countries.

## Methods

### IRB approval

This survey study was deemed exempt by the Office of Human Subjects Research Protections and Institutional Review Board at St. Jude Children’s Research Hospital (Memphis, TN, USA). Voluntary completion of this survey was considered consent to participate in the study.

### Instrument development

A detailed description of the development of the ADAPT survey was previously published (29, 31). Briefly, ADAPT was developed according to the American Association for Public Opinion Research (AAPOR) using WHO guidance on PPC and a review of the literature on physicians’ perceptions of PC and was successfully distributed in Latin America and Eurasia (2, 33–37). For this study, the ADAPT survey went through iterative rounds of review to culturally and contextually adapt to Europe via the SIOPE PPC Working Group. The final survey consisted of 68 items: 65 were close-ended, using a 5-point Likert scale from 5 (“strongly agree”) to 1 (“strongly disagree”), or multiple-choice format, and 3 items were open-ended (**Supplementary Figure S1**).

### Instrument distribution strategy

The ADAPT-E survey was distributed electronically in English via the Qualtrics software platform (38) and shared through the SIOP-Europe listserv from January 27 to February 27, 2023. Physicians of any specialty caring for children with cancer were included in the study. Surveys were completed anonymously, and participation was optional. No overlap in participation occurred between the Eastern European and Central Asian countries involved in the previous ADAPT study in Eurasia (29, 30).

The countries of the participating respondents were categorized into geographic regions, as informed by SIOPE and in alignment with the geographic regions delineated by the Statistics Division of the United Nations (39, 40). Although Turkey and Cyprus are in Asia, their ties to Europe justified their inclusion in the Southern Europe category for our analysis. Specifically, Cyprus is a member of the European Union, and the pediatric hematology-oncology associations of both countries are members of SIOPE.

### Statistical analysis

The statistical analysis in this study mirrored that of previous ADAPT studies (29–33). Demographic data were assessed via descriptive statistics. Only fully completed surveys were included in the final analysis. Of the 68 items in the survey, 15 were categorized as in agreement or disagreement with WHO guidelines for PPC (**Supplementary Table S1**). Physician alignment with WHO guidelines was determined by classifying responses as correct when indicating moderate or strong agreement. An overall alignment score was then calculated for each participant, reflecting the percentage of correct answers out of a maximum possible score of 15.

In the secondary analysis focusing on multiple-choice questions, the responses originally captured on a 5-point Likert scale (ranging from “Never” to “Always”) were collapsed into three categories: “Never/Rarely”, “Sometimes”, and “Often/Always”. This reclassification was done to streamline regional comparisons, employing statistical methods such as Pearson’s χ^2^ or Fisher’s exact test. A *p*-value less than 0.05 was deemed statistically significant. All data summaries and analyses were conducted using SAS software, version 9.4 (SAS Institute) (41).

### Qualitative analysis

Through thematic content analysis, our study aimed to evaluate physicians’ perceptions of the components and role of PPC, their attitudes toward PPC, actions or desires they have for PPC, barriers to delivering PPC in their setting, and optimal timing of PPC initiation.

The qualitative analysis was similar to that in previous ADAPT studies (29–33). The qualitative codebook from previous studies was used, undergoing both inductive and deductive refinement. After conducting an iterative review of the free-text responses, we made minor adjustments to existing codes and added six new codes to the final ADAPT-E codebook (**Supplementary Table S2**). Free-text responses to two of the open-ended questions, “What does palliative care mean to you?” and “If there is a difference between when initial palliative care consultation typically occurs in your setting and what you think is ideal, why do you think this difference exists?”, were qualitatively analyzed using MAXQDA software (42).

Although most survey responses were in English, two were in Hungarian; those responses were translated to English using Google Translate and then confirmed for accuracy by a native Hungarian speaker. Each free-text response served as a unit of analysis for coding.

## Results

### Sample demographics

**Figure 1:**
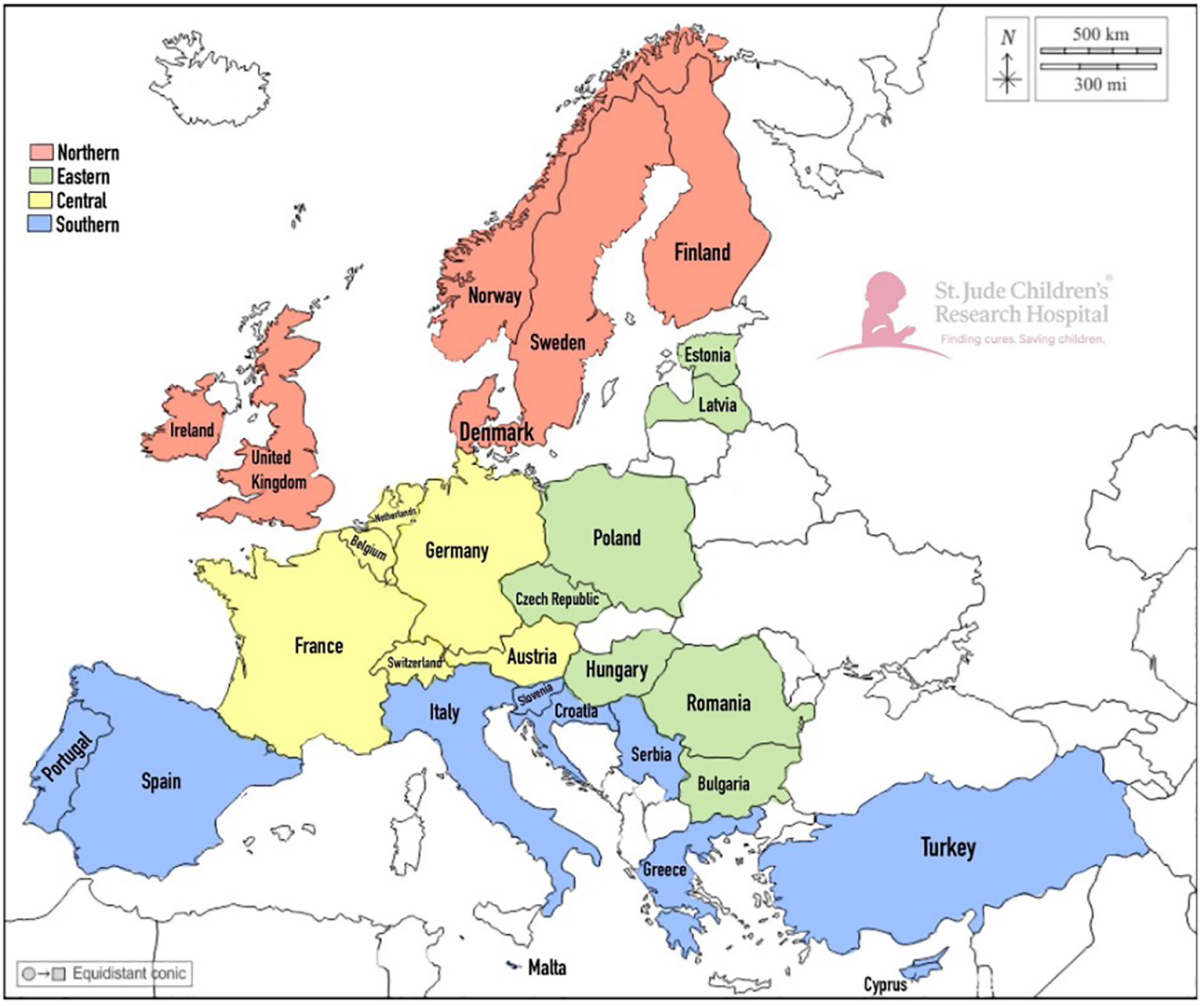
Geographic Distribution of ADAPT Survey Participation by Physicians Caring for Pediatric Patients with Cancer Across European Regions. A map of the European countries represented by the respondents of the survey is shown in the figure. Country colors are associated with the different regions: red is Northern Europe, yellow is Central Europe, green is Eastern Europe, and blue is Southern Europe.

The ADAPT-E survey was completed by 198 physicians from 29 European countries (**Figure 1**, **Table 1**). Another 39 partially completed surveys were removed from analysis. The demographic data showed that most of the respondents were age 35 or older (88.9%), identified as female (67.7%), and had 11 or more years of experience as a physician (85.9%). The most common specialty was pediatric hematology-oncology (74.2%), followed by PPC (9.6%) and general pediatrics (7.1%). Most (86.4%) respondents reported access to PC consultation services. Within this group, the majority (99%) had access to a PC physician and an interdisciplinary PPC team (76%), including psychologists (76%) and nurses (85%). Furthermore, 122 (61.6%) respondents had prior training in PC: 37 (30.3%) completed a certificate course, and 14 (11.5%) completed a dedicated residency or fellowship (**Supplementary Table S7**). Notably, 190 (96.0%) respondents reported having at least 1 patient die in the previous year.

**Table 1:** Demographic characteristics of respondents caring for pediatric patients with cancer to the assessing doctors’ attitudes on palliative treatment survey in Europe.

Characteristic	Respondents, No. (%) N=198
SIOP Europe Member	
Yes No	164 (82.8%) 34 (17.2%)
Country	
Austria Belgium Bulgaria Croatia Cyprus Czech Republican Denmark Estonia Finland France Germany Greece Hungary Ireland Italy Latvia Malta Netherlands Norway Poland Portugal Romania Serbia Slovenia Spain Sweden Turkey United Kingdom Switzerland Missing	4 (2.0%) 10 (5.1%) 3 (1.5%) 3 (1.5%) 1 (0.5%) 4 (2.0%) 2 (1.0%) 1 (0.5%) 2 (1.0%) 8 (4.1%) 12 (6.1%) 7 (3.6%) 14 (7.1%) 2 (1.0%) 26 (13.2%) 1 (0.5%) 1 (0.5%) 16 (8.1%) 5 (2.5%) 3 (1.5%) 9 (4.6%) 8 (4.1%) 7 (3.6%) 2 (1.0%) 19 (9.6%) 5 (2.5%) 3 (1.5%) 13 (6.6%) 6 (3.1%) 1
Age, y	
<35 ≥35	22 (11.1%) 176 (88.9%)
Sex	
Female Male Prefer not to disclose	134 (67.7%) 61 (30.8%) 3 (1.5%)
Primary Medical Specialty	
Pediatric Hematology and/or Oncology Pediatric Palliative Care General Pediatrician Radiation Oncology Pediatric Hematology (Benign) Pediatric Surgery Pediatric Intensive Care Pediatric Anesthesia Adult Palliative Care Other^a^	147 (74.2%) 19 (9.6%) 14 (7.1%) 6 (3.0%) 4 (2.0%) 2 (1.0%) 2 (1.0%) 1 (0.5%) 1 (0.5%) 2 (1.0%)
Primary Institution	
Children’s Hospital General Hospital Cancer Hospital Other^b^	101 (51.0%) 50 (25.3%) 40 (20.2%) 7 (3.5%)
Experience as a physician, y	
0-10 ≥11	28 (14.1%) 170 (85.9%)
Trained in Palliative Care	
Yes No	122 (61.6%) 76 (38.4%)
Access to Palliative Care Consultation	
Yes No	171 (86.4%) 27 (13.6%)
Pediatric Patients Who Died in the Last Year	
0 1-5 ≥6	8 (4.0%) 112 (56.6%) 78 (39.4%)

a Other specialties include Infectious Disease (1) and Psycho-Oncology (1).

b Other institutions include Children’s Cancer Hospital (5), Hospice (1), and Outpatient Clinic (1).

### Alignment with WHO guidelines

Physicians demonstrated relative alignment with WHO guidance for PPC, with a median alignment of 83.4% (range 58.7%-94.4%). Most (94.4%) respondents correctly disagreed with the statement that “children with cancer who receive palliative care die earlier than those who do not”; 91.4% agreed that “palliative care can be integrated with disease-directed therapy,” and 90.7% agreed that “involvement of palliative care during cancer therapy gives greater attention to quality of life and symptom management (e.g. pain, constipation, dyspnea, fatigue).” The three areas of lowest alignment with WHO guidelines included beliefs that “early consultation with palliative care causes increased parental burden and anxiety” (58.7%), “it is difficult to know when a patient with cancer would most benefit from meeting the palliative care team” (60.2%), and “palliative care for children with cancer can be delivered by health care workers of all disciplines, not only by palliative care specialists” (64.8%) (**Supplementary Table S3**).

### Pediatric palliative care in practice and its components

Although some broadly defined PPC as “*accompanying children and their families on a daily basis in order to establish life projects and projects before death, when it is inevitable, in the best possible conditions*,” others focused on specific components, such as “*psychological support to parents, relatives, patient itself.*” A unique theme that emerged from our analysis was the frequent use of the term “holistic”; many respondents described PPC as a “*holistic approach for the care of children, families, and health workers.*” Respondents provided additional insights into their perceptions of PPC through free-text responses (**Supplementary Table S4**).

### Physician comfort

When analyzing physician confidence in evaluating and treating the needs of pediatric patients with serious incurable illnesses, most respondents reported feeling comfortable with addressing the patient’s physical needs (84.4%), as well as the patient and their family’s emotional needs (63.6%). Physicians felt less comfortable assessing and treating spiritual needs (41.9%) or providing grief and bereavement care to the families of patients who die (48.5%). Additionally, 25.3% of respondents reported feeling burdened by their inability to control the suffering of patients at the end of life (EoL) at least some of the time (**Table 2**).

**Table 2:** Physician comfort in addressing the needs of their pediatric patients with cancer in Europe.

Item	No. (%) N=198
I feel confident assessing and treating the physical needs of pediatric patients with serious incurable illness	
Never/Rarely	5 (2.5%)
Sometimes	26 (13.1%)
Often/Always	167 (84.4%)
I feel confident assessing and treating the emotional needs of pediatric patients with serious incurable illness and their families	
Never/Rarely	22 (11.1%)
Sometimes	50 (25.3%)
Often/Always	126 (63.6%)
I feel confident taking care of the spiritual needs of pediatric patients and their families with serious incurable illness	
Never/Rarely	53 (26.8%)
Sometimes	62 (31.3%)
Often/Always	83 (41.9%)
I feel confident providing grief and bereavement care to the families of children who die	
Never/Rarely	42 (21.2%)
Sometimes	60 (30.3%)
Often/Always	96 (48.5%)
I have felt burdened by my inability to control the suffering of children at the end of life	
Never/Rarely	61 (30.8%)
Sometimes	87 (43.9%)
Often/Always	50 (25.3%)

### Ideal vs. actual timing of pediatric palliative care

**Figure 2:**
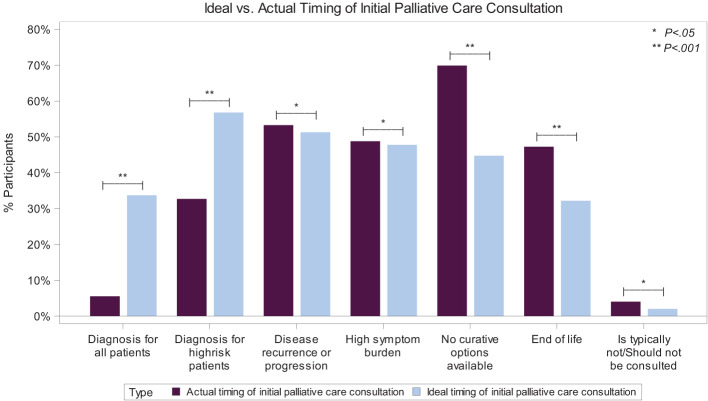
Ideal vs. Actual Timing of Initial Palliative Care Consultation for Pediatric Patients with Cancer. A bar graph showing the significant disparity between the ideal (light blue color) and actual (purple color) timing of the initial pediatric palliative care consultation, depending on specific events. *P <0.05; **P >0.001. Results of 198 physician respondents to the multiple-choice questions asking when initial palliative care consultation for a child with cancer typically occurs in their setting and when they believe is the ideal time it should occur. Participants were asked to “choose all that apply”.

While 34% of physicians believe that, ideally, PPC should be offered at the time of diagnosis for all patients, only 5% said that occurs in their setting (p <0.001). This disparity extends to patients at high risk of relapse or progression: 57% of physicians believe PPC should be integrated at the time of diagnosis, though only 33% have witnessed it in their setting (p <0.001). Conversely, most (70%) stated that PC is integrated in their setting when curative treatments are no longer available, but only 45% believe that timing is ideal (p <0.001). Also, 47% of physicians reported that PC is integrated into patient care at the EoL, but only 32% believe that is the ideal timing (**Figure 2**).

In the free-text responses, physicians elaborated on why the actual and ideal timing for PC integration differed. For example, “*it still feels more natural to start consulting the palliative team in case curative options are limited.*” Another shared that they “*try to find a reason to introduce patients at high risk of relapse to the palliative care team at first diagnosis, for example, if there are troublesome symptoms…*” and “… *would always involve them for patients needing support in symptom control irrespective of prognosis*” (**Supplementary Table S4**).

### Barriers to pediatric palliative care integration

Of the 12 perceived barriers to early integration of PPC included in the survey, eight were indicated as somewhat or extremely important by at least 50% of respondents (**Table 3**). The most frequently perceived barriers included: physician discomfort (82.3%), family resistance (72.2%), limited physician knowledge (71.2%), physician desire to maintain hope (71.2%), and uncertainty about patient prognosis (59.1%). Conversely, the least frequently reported barriers were limited access to opioids (16.7%) and the cost of palliative care education and treatment (17.2%).

**Table 3:** Barriers to the early integration of palliative care for pediatric patients with cancer in Europe.

Barrier	Respondents Indicating Somewhat or Extreme Importance (N=198)
Physician discomfort in raising the topic of palliative care with families	163 (82.3%)
Family resistance to involvement of palliative care	143 (72.2%)
Limited physician knowledge on the role of palliative care	141 (71.2%)
Physician desire to maintain hope	141 (71.2%)
Uncertainty about patient prognosis	117 (59.1%)
Lack of home-based services	108 (54.5%)
Cultural differences between patients/families and physicians	108 (54.5%)
Limited access to palliative care specialists or services	106 (53.5%)
Time constraints of pediatric oncologists during consultation	88 (44.4%)
Differences in languages between patients/families and physicians	84 (42.4%)
Cost of palliative care consultation and treatment	34 (17.2%)
Limited access to opioids	33 (16.7%)

Physicians expanded on the perceived barriers in their settings with their free-text responses. For example, “… *there is still a lack of deep knowledge about pediatric palliative care on the part of other health care providers who take care of the child with cancer. Pediatric palliative care involvement is still unknowingly experienced as a failure by oncologists*,” and how some physicians still have a “*misconception that palliative care is the same as end-of-life care.*” Some respondents also mentioned barriers beyond their control, such as the lack of access, “*Palliative care is not developed, so it is usually not available or it is ‘used’ just for pain management*,” or how PPC integration, “… *depends on the resource in the territory*” (**Supplementary Table S4**).

### Regional analysis

We extended our analysis to compare responses across four European regions: Northern, Southern, Central, and Eastern (**Supplementary Table S5**). While the alignment of physicians’ answers with WHO guidelines across regions was similar (78.1% to 84.2% **Supplementary Table S6**) significant regional differences in several barriers to PPC integration were identified. These included limited physician knowledge of the role of PC (*p* = 0.03), lack of access to home-based services (*p <*0.01), limited access to opioids (*p* = 0.01), and lack of access to PC specialists or services (*p <*0.01) (**Table 4**). While 25.0% of physicians in Central Europe considered the lack of home-based services an important barrier, 76.5% in Eastern Europe and 69.2% in Southern Europe felt this was an important impediment to early integration of palliative care. Additionally, only 7.1% of Central European physicians reported lack of access to opioids as an important barrier, compared to 26.5% in Eastern Europe and 20.5% in Southern Europe. Lastly, 32.1% of physicians in Central Europe stated that the lack of access to PC specialists or services was an important barrier, which was significantly fewer than 73.5% in Eastern Europe and 60.3% in Southern Europe (**Table 4**).

**Table 4:** Important barriers to the early integration of palliative care for pediatric patients with cancer by European Region.

Important Barrier	Northern (%) [N=29]	Central (%) [N=56]	Southern (%) [N=71]	Eastern (%) [N=41]	p-value
Physician knowledge on palliative care	58.6%	66.1%	76.9%	76.5%	0.03
Lack of home-based services	44.8%	25.0%	69.2%	76.5%	<0.01
Limited access to opioids	10.3%	7.1%	20.5%	26.5%	0.01
Limited access to palliative care specialists or services	51.7%	32.1%	60.3%	73.5%	<0.01

## Discussion

Palliative care is considered an essential part of care for serious or life-threatening health conditions. By relieving or preventing symptoms and mitigating the adverse effects of treatment, PC enhances patient’s adherence to their treatment regimens (2). Furthermore, the early integration of PC for patients with life-threatening conditions is an ethical responsibility of clinicians (2). Therefore, understanding physicians’ perceptions of PC is imperative for improving its delivery and implementation.

When we compared our findings with results from previous ADAPT studies, our cohort’s alignment with WHO guidelines was 83.4%, matching the LA cohort (83%) and surpassing the Eurasian cohort (70%) (29, 31). However, further education is warranted, as evidenced by the 40% of our respondents who erroneously stated that “early consultation with PPC causes increased parental burden and anxiety,” and “it is difficult to know when a patient with cancer would most benefit from meeting the PC team.” These findings will help tailor PPC curricula for European physicians to address these misconceptions. Similar initiatives have been successful in Eastern Europe and Central Asia (43).

Additionally, physician knowledge of PPC does not automatically translate into proficient clinical skills or comfort in providing PPC (37). Therefore, we evaluated physicians’ comfort level in addressing patient needs. In our cohort, European physicians were more comfortable addressing the physical and emotional needs of their patients than the LA and Eurasian physicians (29, 31). However, fewer than half were comfortable addressing patients’ spiritual needs or providing grief and bereavement support, which is similar to findings from Eurasia (29). Moreover, family members often feel abandoned by the medical system after their child dies (44–46). Hence, further physician training and education on supporting families after their child’s death is needed. Teaching physicians about grief and bereavement and giving them practical opportunities to interact with and learn from bereaved parents could help alleviate this discomfort (46–48).

Our assessment of perceived barriers to early integration of PPC for pediatric oncology patients revealed three key barriers: physician discomfort about PPC, family resistance, and physician desire to maintain hope. These findings are similar to those from a recent study on PPC availability in pediatric oncology centers across Europe, which identified parental perception, late referrals, and healthcare professionals’ lack of awareness as common barriers (49). These barriers were also identified in the Eurasian and LA ADAPT survey results (30, 32). Despite significant cultural, resource, and linguistic differences across settings, barriers to PPC integration for children with cancer appear to be similar worldwide. Therefore, a global intervention may be adaptable to various contexts and settings. Notably, the access to PC providers, as reported by nearly all respondents in our survey, was much greater than what was reported in LA and Eurasia (29, 31). This discrepancy underscores that access to PC professionals does not fully address the barriers to PPC delivery. There is a global need for concerted efforts to foster an understanding of the essential role and necessity of PPC.

One of the barriers identified in other studies includes the resistance of oncologists to PPC integration due to the strong emotional relationship between the oncologist and their patients and families (18, 19). Concerted education efforts for oncologists and other healthcare professionals caring for children with cancer are needed to increase their comfort in caring for and discussing palliative care with their patients. This provides an opportunity to highlight the unique features of and differences between primary versus specialty palliative care. This training is essential for these professionals to understand and feel comfortable with the elements of primary palliative care, while recognizing when and how specialty palliative care can be integrated into their patients’ treatment (50).

Certain barriers vary by region. For instance, the lack of home-based services was the most common barrier in LA and Eurasia, but less so in Europe. The cost of PC consultation and treatment was identified as a barrier by most physicians in LA and Eurasia, but only by 17% of physicians in Europe (30, 32). These differences support tailoring PC efforts to local needs and account for the unique healthcare systems and cultural contexts in different regions around the world.

European countries exhibit significant diversity, particularly in terms of economic status. Despite all being classified as ‘high-income’ by the World Bank—meaning their gross national income (GNI) per capita exceeds $13,846 USD (51) —the disparities are stark, exemplified by comparing Romania’s GNI per capita of $14,160 to Switzerland’s $90,600 (52). Moreover, we found significant differences in access to PC consultation, home-based services, and opioids across European regions. Southern and Eastern Europe consistently reported greater challenges in these areas, aligning more closely with the patterns seen in Eurasia and LA. This finding highlights the need for creating more equitable access to PPC knowledge, medications, and services (26, 27).

### Study limitations

This study has several limitations. First, the survey was distributed only in English, which may have limited some responses. However, most physicians in Europe do speak English. Second, accurately measuring perceptions is a challenge. Hence the importance of both the quantitative responses regarding perceived barriers and the open-ended free text responses to better understand context. Third, many responses emphasized PPC as a holistic approach to care, but our study specifically focused on the perceptions of physicians. This highlights the need for further research on the perceptions of other healthcare members (e.g. nurses, psychologists, social workers) on the multidisciplinary team about PPC. Fourth, data privacy laws made it impossible to calculate an accurate response rate, which may overestimate the significance of the conclusions drawn. Fifth, distributing the survey through a listserv might have decreased the number of responses due to survey fatigue among members. Lastly, due to the small number of respondents per country, we could not conduct a country-level analysis of the perceptions and barriers.

## Conclusion

This study highlights that throughout Europe, physicians who care for children with cancer understand PPC and are comfortable with the physical and emotional aspects of their patients’ care. Nonetheless, challenges persist, including misconceptions about PC and unease in addressing spiritual needs and providing grief and bereavement support. These areas call for dedicated, concerted educational initiatives and capacity-building efforts. Moreover, despite notable similarities in the barriers identified across regions, considerable differences exist, particularly concerning the lack of PPC specialists, access to opioids, and home-based services. These disparities underscore the need for focused actions to ensure that all children have equitable access to PPC resources regardless of location. The insights gained from this study will inform strategies to improve the quality of life for children with cancer across Europe.

## Data Availability

The original contributions presented in the study are included in the article/**Supplementary Material**. Further inquiries can be directed to the corresponding author.
