# Supplementary material for: Perceptions of physicians caring for pediatric patients with cancer in Europe: insights into the use of palliative care, its timing, and barriers to early integration
Source: Front Oncol. 2024 Oct 23;14:1461668. doi: 10.3389/fonc.2024.1461668 (PMC11538062; doi:10.3389/fonc.2024.1461668)
Supplement: Supplementary file 1 [file DataSheet1.docx]

**Supplemental Materials**

**Perceptions of Physicians Caring for Pediatric Patients with Cancer in Europe: Insights into the Use of Palliative Care, its Timing, and Barriers to Early Integration**

*Ana Lacerda^1,2,3^, *Isabel M. Bravo-Carretero^4^, Bella S. Ehrlich^5,6^, Godwin Job^6^,
María Avilés Martínez^1,7,8^, Ulrike Leiss^1,9^, Georgia Kokkinou^1,10,11^,
Katrin Scheinemann^1,12,13,14^, Finella Craig^1,15^, Kerstin Krottendorfer^1,9^, Meenakshi Devidas^6^,
Justin N. Baker^16^, Asya Agulnik^6^, Michael J. McNeil^6,17^

**Table of Contents**

| **Item** | **Page** |
| --- | --- |
| **Supplemental Figure 1.** ADAPT-E Survey Tool and Reference Sources | 2 |
| **Supplemental Table 1.** ADAPT-E Questions and Alignment to WHO Guidelines | 9 |
| **Supplemental Table 2.** Definitions for the ADAPT-E Qualitative Responses Codebook | 10 |
| **Supplemental Table 3.** Percent Alignment to WHO Guidance Statements | 18 |
| **Supplemental Table 4.** Examples of Physician Responses Used for Qualitative Analysis | 19 |
| **Supplemental Table 5.** European Regions and their Participating Countries | 24 |
| **Supplemental Table 6.** Physicians’ Alignment with WHO Guidelines by European Region | 25 |
| **Supplemental Table 7:** Types of Palliative Care Training | 26 |

**Supplemental Figure 1.** ADAPT-E Survey Tool and Reference Sources

**ADAPT-E Survey**

Demographics

1. Are you an SIOP-Europe member? (Yes/No)
2. Do you treat children with cancer as part of your medical practice? (Yes/No)
3. What is the country in which you currently practice medicine?

| Austria |
| --- |
| Belgium |
| Bosnia and Herzegovina |
| Bulgaria |
| Croatia |
| Cyprus |
| Czech Republic |
| Denmark |
| Estonia |
| Finland |
| France |
| Germany |
| Greece |
| Hungary |
| Iceland |
| Ireland |
| Israel |
| Italy |
| Latvia |
| Lithuania |
| North Macedonia |
| Malta |
| Netherlands |
| Norway |
| Poland |
| Portugal |
| Romania |
| Serbia |
| Slovakia |
| Slovenia |
| Spain |
| Sweden |
| Turkey |
| United Kingdom |
| Switzerland |

1. Your age:

<35 years

35 to 50 years

51 to 65 years

>65 years

1. Your gender:

Female

Male

Other

Prefer not to disclose.

1. Would you consider yourself a religious person? (Religious: the search for significance that occurs within the context of established faith traditions: Judaism, Christianity, Muslim, Hindu, Buddhism, etc.)

Strongly Disagree

Disagree

Uncertain

Agree

Strongly Agree

1. Would you consider yourself a spiritual person? (Spiritual: individual search for meaning and purpose.)

Strongly Disagree

Disagree

Uncertain

Agree

Strongly Agree

1. Please indicate your primary medical specialty:

| Pediatric hematology and/or oncology |
| --- |
| Pediatric anesthesia |
| Pediatric surgery |
| Pediatric palliative care |
| Adult palliative care |
| Adult hematology and/or oncology |
| Adult surgery |
| General pediatrician |
| Pediatric intensive care |
| Radiation oncology |
| Pediatric hematology (benign) |
| Other (describe) |

1. How many years of experience in practice do you have since graduating medical school?

0-5 years

6-10 years

11-15 years

16-20 years

21+ years

1. Concerning the primary institution in which you work, indicate if it is a:

General Hospital

Children’s Hospital

Cancer Hospital

Other (describe)

1. Have you received any formal training in palliative care?

Yes

No

If yes, check all that apply:

Continuing Medical Education (or postgraduate course without official

certification)

Certificate course

Medical School or Post-graduate Rotation

Undergraduate/Medical School Course

Masters in Palliative Care

Residency or Fellowship in palliative care

Other (please describe)

12. Do you have access to a pediatric palliative care expert for consultation in your practice?

(Yes/No)

If yes, please check the type(s) of palliative care experts available:

Physician

Nurse

Social worker

Psychologist

Other (please describe)

13. How many pediatric patients in your care (less than 18 years old) died in the last 12 months?

0 patients

1-5 patients

6-10 patients

11-20 patients

21+ patients

14. What does palliative care mean to you?

Perspectives

Please rate the extent to which you agree with the following statements about pediatric oncology patients receiving palliative care. Please check one box per line:

Strongly Disagree

Somewhat Disagree

Neutral

Somewhat Agree

Strongly Agree

15. It is difficult to know when a patient with cancer would most benefit from meeting the

palliative care team.

16. Quality of life is often overlooked in the face of cancer-directed treatment.

17. Children with advanced and incurable cancer often suffer at the end-of-life.

18. Early consultation with palliative care causes increased parental burden and anxiety.

19. Palliative care is perceived by parents as meaning the end of life is near and that the

oncologist will ‘give up’ on their child.

20. Palliative care can be integrated with disease-directed therapy.

21. Involving palliative care suggests the oncologist has failed in the mission to cure the patient.

22. There are situations where it is in a dying child’s best interest to remove mechanical

ventilation, if in alignment with the family’s wishes.

23. Palliative care for children with cancer can be delivered by health care workers of all

disciplines, not only by palliative care specialists.

24. Palliative care is synonymous with “end-of-life” care.

25. Involvement of palliative care during cancer therapy gives greater attention to quality of life

and symptom management (e.g. pain, constipation, dyspnea, fatigue).

26. Involvement of palliative care undermines the role of the pediatric oncologist as the

physician in charge of patient care.

27. Children with cancer who receive palliative care die earlier than those who do not.

28. Early integration of palliative care for all children diagnosed with cancer would decrease

patient suffering.

29. Early integration of pediatric palliative care with cancer care would improve interdisciplinary

communication.

30. Palliative care is incompatible with curative care.

31. Involving the palliative care team early has negative effects on the relationship between the

oncologist and the patient and their family.

32. Palliative care is appropriate at any stage of treatment in a child with high-risk cancer.

33. Administering opioids to patients in pain hastens death due to respiratory depression.

34. In my setting, physicians typically continue to recommend cancer-directed treatment for

their patients with incurable oncological disease, even when that treatment is ineffective or unlikely to prolong their patient’s life.

35. In my setting, doctors generally feel confident taking care of the physical needs of pediatric

patients with a serious incurable illness.

36. In my setting, doctors generally feel confident taking care of the emotional needs of pediatric

patients with a serious incurable illness.

37. In my setting, doctors generally feel confident taking care of the spiritual needs of pediatric

patients and their families with a serious incurable illness.

38. In my setting, doctors generally feel confident providing grief and bereavement care to the

families of children who die.

39. Greater emphasis on palliative care education for doctors and health care professionals is an

important step in improving access to palliative care.

40. I wish to have more education on how to provide palliative care to my patients.

Individual Experience

Please rate the frequency of the following statements about your individual experiences with

pediatric oncology patients. Please check one box per line:

Never

Rarely

Sometimes

Often

Always

41. In my setting of practice, palliative care consultation is available when I feel it is needed for a

child with cancer.

42. In my setting, I have felt that involvement of palliative care has occurred too late in the

treatment of a child with cancer.

43. In my setting, I have acted against my conscience by providing aggressive treatment to a

pediatric oncology patient with advancing disease.

44. I feel confident assessing and treating the physical needs of pediatric patients with serious

incurable illness.

45. I feel confident assessing and treating the emotional needs of pediatric patients with a serious

incurable illness and their families.

46. I feel confident taking care of the spiritual needs of pediatric patients and their families with

a serious incurable illness.

47. I feel confident providing grief and bereavement care to the families of children who die.

48. I have felt burdened by my inability to control the suffering of children at the end of life.

Multiple choice

Please choose all that apply for every option:

49. The role of palliative care in the treatment of children with cancer is (please choose all that apply):

- To aid in reducing pain and suffering related to disease and/or treatment

- To provide psychological support to the patient and their family

- To provide spiritual support to the patient and their family

- To aid in family decision-making around treatment options

- To help clarify the goals of care of the patient and their family

- To help communicate bad news to the patient and their family

- To aid in communication between the patient, their family, and medical teams

- To assist with transitions from the hospital to hospice or home at the end of life

- Other: Please describe

50. When does initial palliative care consultation for a child with cancer typically occur in your setting? (please choose all that apply):

- At the time of cancer diagnosis for all patients

- At the time of cancer diagnosis for patients at high risk of relapse or progression

- At the time of disease relapse or progression

- At the time of complex or high symptom burden (e.g. pain, suffering)

- When there are no longer curative therapeutic options available

- At the end of life

- Palliative care is typically not consulted for children with cancer (because it is not

necessary or not available)

51. Assuming unlimited resources, when do you think is the ideal timing of initial palliative care

consultation for a child with cancer? (please choose all that apply):

- At the time of cancer diagnosis for all patients

- At the time of cancer diagnosis for patients at high risk of relapse or progression

- At the time of disease relapse or progression

- At the time of complex or high symptom burden (e.g. pain, suffering)

- When there are no longer curative therapeutic options available

- At the end of life

- Palliative care consultation is never necessary in pediatric cancer care

52. If there is a difference between when initial palliative care consultation typically occurs in

your setting and what you think is ideal, why do you think this difference exists?

Barriers

Please rate the extent to which you feel the following statements represent barriers to early

integration of palliative care for pediatric oncology patients. Please check one box per line:

Extremely Unimportant Barrier

Somewhat Unimportant Barrier

Neither Important nor Unimportant Barrier

Somewhat Important Barrier

Extremely Important Barrier

53. Limited physician knowledge on the role of palliative care

54. Physician discomfort in raising the topic of palliative care with families

55. Physician desire to maintain hope

56. Uncertainty about patient prognosis

57. Family resistance to the involvement of palliative care

58. Time constraints of pediatric oncologists during consultation

59. Lack of home-based services

60. Limited access to opioids

61. Limited access to palliative care specialists or services

62. Cost of palliative care consultation and treatment

63. Cultural differences between patients and their families with their physicians

64. Language differences between patients and their families with their physicians

65. Are there barriers to early integration of palliative care for pediatric oncology patients not

listed above? If yes, please identify the barriers and rate their importance below. If no additional barriers exist, go to the next question.

66. Do you have any additional comments or concerns regarding palliative care for children and

adolescents with cancer?

**Supplemental Table 1: ADAPT-E Questions and Alignment with WHO Guidelines**

| **Does the question align with WHO ADAPT statements?** | |
| --- | --- |
| **Survey Item** | **WHO Alignment** |
| 17-Children with advanced and incurable cancer often suffer at the end-of-life | Agree |
| 20-Palliative care can be integrated with disease-directed therapy | Agree |
| 23-Palliative care for children with cancer can be delivered by health care  workers of all disciplines, not only by palliative care specialists | Agree |
| 25-Involvement of palliative care during cancer therapy gives greater attention  to quality of life and symptom management (e.g. pain, constipation,  dyspnea, fatigue) | Agree |
| 28-Early integration of palliative care for all children diagnosed with cancer  would decrease patient suffering | Agree |
| 29-Early integration of pediatric palliative care with cancer care would improve  interdisciplinary communication | Agree |
| 32-Palliative care is appropriate at any stage of treatment in a child with high-  risk cancer | Agree |
| 15-It is difficult to know when a patient with cancer would most benefit from  meeting the palliative care team | Disagree |
| 18-Early consultation with palliative care causes increased parental burden and  anxiety | Disagree |
| 21-Involving palliative care suggests that the oncologist has failed in the  mission to cure the patient | Disagree |
| 24-Palliative care is synonymous with “end-of-life” care | Disagree |
| 26-Involvement of palliative care undermines the role of the pediatric oncologist  as the physician in charge of patient care | Disagree |
| 27-Children with cancer who receive palliative care die earlier than those who  do not | Disagree |
| 30-Palliative care is incompatible with curative care | Disagree |
| 31-Involving the palliative care team early has negative effects on the  relationship between the oncologist and the patient and their family | Disagree |

**Supplemental Table 2. Definitions for the ADAPT-E Qualitative Responses Codebook**

**ADAPT-E Perceptions of Palliative Care**

[Used for Question 14: “What does palliative care mean to you?”]

| **Category** | **Code** | **Definition** |
| --- | --- | --- |
| Component/Role of Palliative Care |  |  |
|  | Accompany | Refers to the role of the palliative care provider/team to accompany the patient and their family in experiencing a chronic or life-threatening illness. |
|  | Psychosocial Support | Reference to palliative care as any general psychological and/or social support, assistance, and care provided by the physician or palliative care team to the patient and/or family. This includes any mention of “mental” or “social” support and any psychological support provided to families around the dying process, either at the end-of-life or after the passing of the patient. This code also includes grief, bereavement, and mourning support.  Does NOT include terms that describe a patient’s emotional state ( “emotional,” “moral.” Code under “Patient Quality of Life”). |
|  | Patient Quality of Life | Reference to palliative care as the quality of life of the patient, including any mention of “suffering,” or “comfort.” This includes any mention of “maximum” benefit and “individualized,” “personalized,” “specific,” “customized” care to the patient, and “patient QoL,” “alleviating,” “relieving,” “easing,” “facilitating,” “improving,” and “ameliorating.”  Do NOT code vague terms such as “caring,” “helping,” or “supporting” if there is no context. |
|  | Medical Care | Reference to palliative care as medical treatment or medical care, which includes any mention of “procedures,” “interventions,” “treatment,” “medical care,” “radiotherapy,” “chemotherapy,” “nutrition,” or medicine (“opioids,” “analgesia”). This also includes body processes (“functions,” “vitals”).  Does NOT include management of symptoms or caring for the “physical condition”, implying symptoms such as “pain” (Code under “Symptom Management”). |
|  | Symptom Management | Reference to palliative care as symptom therapy or pain management, rehabilitation, and improvement of “pain” or the “physical condition.” This includes any mention of “maintenance.”  Does NOT include any mentions of “suffering” only (Code under “Patient Quality of Life”) or any medical care or treatment NOT directed at symptoms (Code under "Medical Care"). |
|  | Life Extension | Reference to palliative care as involving the “lengthening”, “prolongation”, “extension” of life. Does NOT include references of solely lengthening moments of high quality of life (Code under “Patient Quality of Life”) or symptom management (Code under “Symptom Management”) within the same course of a patient’s life. |
|  | End-of-Life Care | References to palliative care as “end-of-life care”, “death”, or “passing away”. This includes mentions of easing the process of death and of normalization and acceptance of death for the patient and/or family (any mention of “death” and “passing away”). DO include “until end of life” when implying that end of life is a component of palliative care. |
|  | Religious/ Spiritual Care | Reference to palliative care as the provision of spiritual or religious support to the patient and/or family. |
|  | Teamwork | Reference to palliative care as an “interdisciplinary” and/or “multidisciplinary” communication within one institution or collaboration between institutions. This includes specific references to the involvement of nurses, psychologists, or different specialists. DO include any mention of the team facilitating the transition from the hospital to hospice or home at the end-of-life of the patient. |
|  | Communication with Patient and Family | Reference to palliative care as any communication delivered by the physician to the patient and/or family, whether it be the communication of bad news, aid in family decision-making around treatment options, or helping clarify the goals of care of the patient and their family. This includes mention of the LACK of communication with the patient’s family on these topics.  Does NOT include refusal of care by the patient’s family in response to provider communication (Code under “Society Attitudes”). |
|  | Holistic Approach | References to palliative care as being a “holistic,” “integrative,” “multimodal,” “comprehensive” way to take care of patients. This also includes any mention of “global” or “optimal” care or treatment “in all other ways.” |
|  | Patients with Life-Limiting Disease | References to palliative care as a service for patients who have a “chronic,” “life-limiting,” “life-threatening,” “degenerative,” “potentially fatal,” “fatal,” or “oncologic” disease, as well as having “cancer” or a “serious”, “terminal stage” illness. |
|  | Support/Care/ Help for Patients and their Family | References to palliative care as “supporting,” “caring,” or “helping” the patient and their families with or without a specific time point (before, during, or after diagnosis or treatment). DO include any mention of the quality of life of the patient’s family and addressing the “wishes,” “needs,” or “problems” of the patient and their family. |
|  | Hospice and Home Health Care | References to palliative care as having a component of “hospice,” “at home” care, “more time at home” or being “away from the hospital.” |
| Timing of Palliative Care |  |  |
|  | At Diagnosis | References the timing of palliative care involvement at the time of diagnosis. Refers to a specific time point when palliative care is integrated. Does NOT imply exclusivity with curative treatment. |
|  | Disease Progression | References any mention of specific time points of palliative care integration or involvement when a patient is given a poor prognosis, disease relapse or progression, or high symptom burden (e.g. “pain”, “suffering”).  Does NOT include at diagnosis (Code under “At Diagnosis”), at the end-of-life (Code under “End-of-life/Terminal Stages”), or after treatment options are exhausted (Code under “No Other Options Available”).  This does NOT imply exclusivity with curative treatment or include references to palliative care as an individualized approach (Code under “Patient Quality of Life”) or based on need (Code under “No Other Options Available”), unless there are further specifications about timing in the course of treatment or the life of the patient. |
|  | Integration with Treatment | References to palliative care being integrated concurrently with treatment, with NO specific mention of time points, such as at diagnosis (Code under “At Diagnosis”) or disease relapse/high symptom burden (Code under “Disease Progression”). This includes key phrases such as “early integration” and any mention of palliative care being integrated before diagnosis, during treatment, or “all the way through” a patient’s disease course. Note: this IS mutually exclusive with other timing codes. |
|  | No Other Options Available | References the timing of palliative care involvement as conditional upon patient unresponsiveness to treatment and/or other treatment options being unavailable or “exhausted” (“treatment is failing,” “no options available,” “did everything possible”). Refers to a specific time point when palliative care is integrated or involved in a patient’s disease course. Often there is an implication of mutual exclusivity between curative therapy and symptomatic treatment/palliative care. DO include any mention of “incurable” disease/patients under this code. |
|  | End of Life/Terminal Stages | References the specific time points of palliative care integration or involvement at the end of life of a patient, including terms such as “last resort,” “last days of life,” or “final landing.” This also includes grief and bereavement support after death.  Any reference to care delivered to terminal patients, with no indication of previous exposure, is coded (e.g. “to improve the quality of life of end-stage patients”). However, do NOT code words or phrases like “until end of life”, as it leaves open-ended when palliative care was initiated (Code under “End-of-life Care”).  Note: This will often be double-coded with “end-of-life care.” |
| Positive Attitudes |  |  |
|  | Celebration of Life | Refers to any comment on palliative care or hospice as a celebration of life for the patient and/or their family. Keywords include “house of life”, “better”, or “decorating” life. |
|  | Compassion and Love | Refers to the provision of compassion and love to the patient and/or family. Keywords include “care”, “love”, “hope”, or “empathy.” |
|  | Necessary Service | Refers to palliative care as a mandatory service for any indicated population. Keywords include “mandatory,” “crucial,” “integral,” “necessary,” “need,” or “important.” Also includes references to palliative care as a patient’s right. DO include any mention of palliative care being a “free” or “government-funded” service. |
|  | Life/Death with Dignity | References to palliative care or hospice using any mention of the word “dignity” or “worth” over the course of the patient’s life and/or death. |

**ADAPT-E Actions, Barriers, and Timing of Pediatric Palliative Care**

[Used for Question 52: “If there is a difference between when initial palliative care consultation typically occurs in your setting and what you think is ideal, why do you think this difference exists?”.]

| **Category** | **Code** | **Definition** |
| --- | --- | --- |
| Actions/ Desires |  |  |
|  | Satisfied with Status Quo | Refers to any opinions that the status quo is currently satisfactory or that palliative care should not exist. |
|  | Need for Systemic Change | References to desires to integrate or improve palliative care services in the future (e.g. provision of “individualized care based on need”). This refers to provider perspectives of what should be or what they hope should happen in the future, and who should be responsible for palliative care, if this implies training more palliative care specialists. Note: this is different from a right to have palliative care (Code under “Necessary Service”), and NOT ALL statements using the word “should” indicate desire for change, but rather a factual statement. |
| Barriers |  |  |
|  | Physician Attitudes | Refers to any physician attitudes that serve as a barrier to early integration of palliative care. This includes provider lack of education, awareness, or understanding about palliative care, or hesitation to administer drugs (e.g. pain medication). This also includes provider feelings of ambition, hope, fear, failure, or burnout that prevent earlier referral and perceptions that there are no opportunities to include other specialists or other challenges with interdisciplinary communication. It also includes provider choices to not communicate diagnoses or prognoses with the patient or their family but does NOT include provider perceptions of parent unreadiness (Code under “Family Attitudes”). Does NOT include health systems barriers such as lack of drug access (Code under “Systemic”) or lack of palliative care experts (Code under “Access”). Note: This includes any mention of education. |
|  | Family Attitudes | Refers to family attitudes that serve as a barrier to early integration of palliative care. This includes mentions of the family’s lack of education, misperceptions, or awareness. This further includes family readiness or hope that leads to refusal of palliative care. Also includes mentions of difficulty communicating with the family, as well as the family not communicating with their child. |
|  | Society Attitudes | Refers to the attitudes of the greater community or society that serve as a barrier to early integration of palliative care. This includes mentions of society misperceptions, stigma, or greater “culture.” |
|  | Institutional | Refers to any structural barriers within the institution (e.g. hospital, organization, center) that prevent early integration of palliative care, but NOT on a larger legislative/federal health systems level (e.g. hospital collaboration, bed shortage, infrastructural limits within the hospital; Code under “Systemic”). This further includes understaffing, heavy oncologist workload, lack of administrative support, or administrative ignorance/lack of education. |
|  | Systemic | Refers to larger systemic/federal level (health systems) barriers that prevent early integration of palliative care. This includes a lack of organized education on a health systems level without mention to specific individuals (e.g. “the opportunity to specialize”), poor legislation, lack of accepted standards, lack of funding, strict drug regulations, lack of drug access/procurement, or poor infrastructure/distribution of resources across regions. Includes any mention of laws or policies and any general mention of resources (e.g. financial). Does NOT include references to individual lack of training or choices to not administer drugs (Code under “Physician Attitudes”). |
|  | Access | Refers to access to palliative care specialists, services, and consults that serve as a barrier to its integration, including the existence or access to trained specialists or a palliative care service/team (e.g. “no available care,” “not enough doctors”). Does NOT include references to access to overworked general providers/oncologists (e.g. “need to develop palliative care in all areas”; Code under “Institutional”) |
|  | Misconception that Palliative Care = End-of-Life Care | Refers to any mention that the term “palliative care” is synonymous with end-of-life care, and how this can limit physician, patient, and/or family’s willingness/readiness to integrate palliative care into the patient’s care. |
| Timing of Palliative Care |  |  |
|  | At Diagnosis | Any references to the timing of palliative care involvement at the time of diagnosis. Refers to a specific point in time when palliative care is integrated. Does NOT imply exclusivity with curative treatment. |
|  | Disease Progression | Any references to specific time points of palliative care integration or involvement that are NOT at diagnosis (Code under “At Diagnosis”), at the end-of-life (Code under “End-of-Life/Terminal Stages”), or after treatment options are exhausted (Code under “No Other Options Available”). This includes involvement given poor prognosis for patients with high-risk disease, disease relapse or progression, and high symptom burden (“pain”, “suffering”). This does NOT imply exclusivity with curative treatment or include referencing palliative care by using an individualized approach or an on-need basis unless there are further specifications about timing in the course of treatment or the life of the patient. |
|  | Integration with Treatment | Any references to palliative care to be integrated concurrently with treatment, with NO specific mention of any point in time, such as at diagnosis (Code under “At Diagnosis”) or disease relapse/high symptom burden (Code under “Disease Progression”). This also includes key phrases such as “early integration.” Note: This IS mutually exclusive with other timing codes. |
|  | No Other Options Available | This references the timing of palliative care involvement as conditional upon patient unresponsiveness to treatment and/or other treatment options being unavailable and “exhausted” (e.g. “treatment is failing,” “no options available”). Refers to a specific time point when palliative care is integrated or involved in a patient’s disease course. Often there is an implication of mutual exclusivity between curative therapy and symptomatic treatment/palliative care. DO include any mention of “incurable” disease. |
|  | End-of-Life/ Terminal Stages | Any references to the specific points of palliative care integration or involvement at the end-of-life of a patient, including terms such as “last resort,” “last days of life,” or “final landing.” This also includes grief and bereavement support after death. Any reference to care delivered to terminal patients, with no indication of previous exposure, is coded (e.g. “to improve quality of life of end-stage patients”). However, do NOT code words “until end of life,” as it leaves open-ended when palliative care was initiated (Code under “End-of-Life Care”). Note: This will often be double-coded with “End-of-Life Care.” |
|  | Palliative Care Hesitance Leads to Late Referral | Refers to any mention that palliative care is integrated late, as well as wishes that palliative care be integrated earlier in a patient’s care. This includes any mention of having to postpone palliative care integration due to misperceptions that it is too “early” or “soon.” |

**Supplemental Table 3.** Physicians’ Percent Alignment to WHO Guidance Statements

| **Statements Assessing Alignment with WHO Guidance** | **Percent Alignment (%)** |
| --- | --- |
| 18- Early consultation with palliative care causes increased parental burden and anxiety | 58.7 |
| 15- It is difficult to know when a patient with cancer would most benefit from meeting the palliative care team | 60.2 |
| 23- Palliative care for children with cancer can be delivered by health care workers of all disciplines, not only by palliative care specialists | 64.8 |
| 17- Children with advanced and incurable cancer often suffer at the end-of-life | 71.1 |
| 28- Early integration of palliative care for all children diagnosed with cancer would decrease patient suffering | 73.7 |
| 31- Involving the palliative care team early has negative effects on the relationship between the oncologist and the patient and family | 78.7 |
| 32- Palliative care is appropriate at any stage of treatment in a child with high-risk cancer | 78.7 |
| 26- Involvement of palliative care undermines the role of the pediatric oncologist as the physician in charge of patient care | 82.7 |
| 24- Palliative care is synonymous with “end-of-life” care | 85.8 |
| 30- Palliative care is incompatible with curative care | 85.8 |
| 29- Early integration of pediatric palliative care with cancer care would improve interdisciplinary communication | 86.8 |
| 21- Involving palliative care suggests that the oncologist has failed in the mission to cure the patient | 86.8 |
| 25- Involvement of palliative care during cancer therapy gives greater attention to quality of life and symptom management (e.g. pain, constipation, dyspnea, fatigue) | 90.7 |
| 20- Palliative care can be integrated with disease-directed therapy | 91.4 |
| 27- Children with cancer who receive palliative care die earlier than those who do not | 94.4 |

**Supplemental Table 4: Examples of Physician Responses Used for Qualitative Analysis**

**ADAPT-E Perceptions of Pediatric Palliative Care Examples**

[Examples of Answers for Question 14: “What does palliative care mean to you?”.]

| **Category** | **Code** | **Sample Response** |
| --- | --- | --- |
| Component/Role of Palliative Care |  |  |
|  | Accompany | “It is about accompanying children and their families on a daily basis in order to establish life projects and projects before death, when it is inevitable, in the best possible conditions.” |
|  | Psychosocial Support | “Psychological support to parents, relatives, patient itself.” |
|  | Patient Quality of Life | “Overall care with consideration for quality of life/terminal care in every stage of the patient's ilness/cancer journey aligned with patient's way of life, with optimal holistic support.” |
|  | Medical Care | “Providing supportive and medical care for terminally ill children.” |
|  | Symptom Management | “Palliative care is a holistic care of children with potentially fatal illness focusing on providing relief of symptoms and quality of life since the diagnosis taking in account all aspects, physical, psychological social and spiritual.” |
|  | Life Extension | “Add life to the time the child have than prolong the time.” |
|  | End-of-Life Care | “Current UK usage - care of children who will, in all probability, die young of their disease.” |
|  | Religious/ Spiritual Care | “Palliative care is a holistic way to care patient and his/her family. It covers symptom care, hopefully with an early integration and psychosocial as well as spiritual support.” |
|  | Teamwork | “A holistic way of addressing the problems and needs of patients and their families, with the greatest possible medical evidence and the highest excellence, developed by an interdisciplinary and multidisciplinary team (doctors, nurses, therapists, psychologists, social workers, volunteers, etc.) that works in a network, respecting their autonomy, their customs, their ties, etc. in the best interest of the patient” |
|  | Communication with Patient and Family | “Support to families with and during tough treatment decissions. To help in a process to anable passing away of a child in any ways a family needs and wants to.” |
|  | Holistic Approach | “An holistic approach for the care of children, families and health workers” |
|  | Patients with Life-Limiting Disease | “Palliative care means optimal care for patients without chance of cure and with chronic degenerative diseases.” |
|  | Support/Care/ Help for Patients and Family | “Maximum support for the sick child and his family.” |
|  | Hospice and Home Health Care | “More time at home/ in an environment where the patient is more happy.” |
| Timing of Palliative Care |  |  |
|  | At Diagnosis | “Palliative care starts at the point of diagnosis.” |
|  | Disease Progression | “Taking care since the beginning of the phase when the disease has relapsed or is refractory.” |
|  | Integration with Treatment | “All the care that can be provided to a child and his family to increase their quality of life while being treated for a life-threatening or life-limiting disease.” |
|  | No Other Options Available | “this is a continum of care when curative treatment are not available anymore. this might include  - symptoms management  - palliative anti-cancer treatment  - psycho-social support for child and familly  - pursuing project  - hospital and home care” |
|  | End-of-Life/Terminal Stages | “providing comfort and quality of life at the end of life with support for patient and family members” |
| Positive Attitudes |  |  |
|  | Celebration of Life | “Offering the best quality of care in the palliative phase of life. adding 'good' days to the rest of their life. |
|  | Compassion and Love | “Empathy, sympathy, positive approach, prayer can help very much.” |
|  | Necessary Service | “One of the cornerstones of care for many of our patients.” |
|  | Life/Death with Dignity | “A humane and dignified send-off for the child, when the medical profession did everything possible |

**ADAPT-E Actions, Barriers, and Timing of Pediatric Palliative Care Examples**

[Examples of Answers for Question 52: “If there is a difference between when initial palliative care consultation typically occurs in your setting and what you think is ideal, why do you think this difference exists?”.]

| **Category** | **Code** | **Sample Response** |
| --- | --- | --- |
| Actions/ Desires |  |  |
|  | Satisfied with Status Quo | “not all standard risk patients require palliative or specialist symptom care management. Oncologist are skilled at managing standard side effects from cancer directed therapy and the cancer itself. Palliative care does not need to do all symptoms this would undermine and deskill the oncology team. In regard to the spiritual and psychological - this is not just the palliative care domain- this should be offered to all children with cancer- so I don't think palliative care should offer this- this should be part of the standard cancer service provision for all.” |
|  | Need for Systemic Change | “There is no specified PC team however the treating team tries to cover the PC needs (there is a need for a specified taem)” |
| Barriers |  |  |
|  | Physician Attitudes | “Because there is still a lack of deep knowledge about pediatric palliative care on the part of other health care providers who take care of the child with cancer. Pediatric palliative care involvement is still unknowingly experienced as a failure by oncologists.” |
|  | Family Attitudes | “Time of such a consultation should depend on each case. There are important psychological obstacles - mainly families of patients to whom we would propose a palliative consultation before all curative therapeutic options are available, would often see it as a ‘bad omen’ or a sign that the medical team has already given up on a patient and doomed the child ‘incurable’.” |
|  | Society Attitudes | “Taboo, another added burden for the family, ignorance about palliative care and its role, both for doctors and society.” |
|  | Institutional | “Different physicians have different perspective and lack of a common recommendation at the hospital.” |
|  | Systemic | “It depends on the resource in the territory” |
|  | Access | “Palliative care is not developed, so it is usually not available or it is ‘used’ just for pain management” |
|  | Misconception that Palliative Care = End-of-Life Care | “Misconception that palliative care is the same as end-of-life care.” |
| Timing of Palliative Care |  |  |
|  | At Diagnosis | “Palliative consultation and support since the diagnosis.” |
|  | Disease Progression | “I always try to find a reason to introduce patients at high risk of relapse to the palliative care team at first diagnosis, for example if there are troublesome symptoms, and I would always involve them for patients needing support in symptom control irrespective of prognosis.” |
|  | Integration with Treatment | “Palliative care consultation typically occurs in the last part of the treatment/life at progression/relapse but ideally for patients with aggressive disease at high risk I think it could be better to start with palliative care simultaneous to oncological treatment at diagnosis.” |
|  | No Other Options Available | “It still feels more natural to start consulting the palliative team in case curative options are limited.” |
|  | End-of-Life/Terminal Stages | “Palliative care consultation typically occurs in the last part of the treatment/life.” |
|  | Palliative Care Hesitance Leads to Late Referral | “Ideal should be at diagnosis but tipically occurs later.” |

**Supplemental Table 5: European Regions and their Participating Countries**

| **European Region** | **Countries** |
| --- | --- |
| Northern | Denmark, Finland, Ireland, Norway, Sweden, United Kingdom |
| Central | Austria, Belgium, France, Germany, Netherlands, Switzerland |
| Southern | Croatia, Cyprus, Greece, Italy, Malta, Portugal, Serbia, Slovenia, Spain, Turkey |
| Eastern | Bulgaria, Czech Republic, Estonia, Hungary, Latvia, Poland, Romania |

**Supplemental Table 6: Physicians’ Percent Alignment to WHO Guideline Statements by European Region**

| **European Region** | **Median (%)** | **Range (%)** |
| --- | --- | --- |
| Northern | 79.2 | 58.3-95.8 |
| Central | 80.0 | 56.4-98.2 |
| Southern | 84.2 | 51.3-94.7 |
| Eastern | 78.1 | 43.8-96.9 |

**Supplemental Table 7: Types of Palliative Care Training**

| **Types of Palliative Care Training** | N=122 (61.6%) |
| --- | --- |
| Continuing Medical Education (or postgraduate course without official certification) | 68 (55.7%) |
| Certificate course | 37 (30.3%) |
| Medical school or postgraduate rotation | 24 (19.7%) |
| Masters in Palliative Care | 19 (15.6%) |
| Undergraduate / medical school course | 14 (11.5%) |
| Residency or Fellowship in Palliative Care | 14 (11.5%) |
| *Other | 1 (0.008%) |

*Other: Clinical practice

Respondents could select more than one option with regards to training.
